# Randomized comparison of W.A.R.A. (Wiring Affect with ReAttach) versus distraction: A pilot study assessing the efficacy of an ultrafast transdiagnostic intervention

**DOI:** 10.1016/j.heliyon.2020.e04660

**Published:** 2020-08-09

**Authors:** Paula Weerkamp-Bartholomeus, Donatella Marazziti, Edward Chan, Ashutosh Srivastava, Therese van Amelsvoort

**Affiliations:** a Department of Psychiatry and Neuropsychology, School for Mental Health and Neuroscience, Maastricht University, Maastricht, the Netherlands; b ReAttach Therapy International Foundation, Voerendaal, the Netherlands; c Dipartimento di Medicina Clinica e Sperimentale, Section of Psychiatry, University of Pisa, Italy; d International Psychology Centre, Kuala Lumpur, Malaysia; e Bharatiya Counselling Psychology Association, New Delhi, India; f Psyuni Trust, Lucknow, India

**Keywords:** Psychology, Clinical psychology, W.A.R.A., Distraction, ReAttach, Affect regulation, Emotion regulation, Neuropsychiatric Disorders

## Abstract

**Background:**

Generally, neuropsychiatric patients share different symptoms across nosological categories, such as, amongst other, psychological distress, mood alterations, anxiety, and self-regulation disturbances.

ReAttach is a novel psychological intervention with its key elements being external affect and arousal regulation, stimulation of multiple sensory processing, conceptualization, affective mentalization, and associative memory processing. ReAttach has been hypothesized to be effective in reducing symptom severity in different psychiatric conditions. Given the limited information currently available, the present study aimed to investigate the effect of main ReAttach elements called “Wiring Affect with ReAttach” (W.A.R.A.) on negative affect (N.A.), and to compare it with “Distraction,” another well-established affect-regulating strategy.

**Methods:**

We used a single-blind, randomized controlled crossover equivalence design to compare the efficacy on N.A. regulation of W.A.R.A. versus Distraction in 101 patients with different neuropsychiatric disorders.

**Results:**

The results showed a significant difference (p < 0.001) in response to W.A.R.A. vs. Distraction, with W.A.R.A. being significantly more effective in regulating N.A., with a large effect size (dRMpooled = 2.38) and a high probability (95%) of success.

**Limitations:**

The heterogeneity of the study population makes generalization and clear recommendations for specific patient groups difficult. The Numeric Rating Scale might have prevented detection of increased N.A. when the baseline scores were high. More in-depth research is needed to explore the W.A.R.A. technique and the extent of confounding variables such as the placebo effect.

**Conclusions:**

The findings suggest that W.A.R.A. may be an effective, accessible, and brief intervention reducing negative affect. Although premature, these first results are encouraging.

## 1 Introduction

According to the World Health Organization (W.H.O.), in the last years, 25 % of European adults have been suffering from mental health problems, especially those involving affective disorders (W.H.O., 2015). Affect is an individual sensory experience that might serve as an essential factor within a wide range of psychological processes. Affective states influence the individual's physical, cognitive, emotional, and behavioral functioning (Aldao, 2010; Espeleta, 2019; Schultheis, 2019; Weiss, 2019). Arousal regulation, sensory integration, and conceptualization can be regarded as requirements for negative affect (N.A.) regulation.

During N.A. regulation, unpleasant internal and external sensory stimuli need to be integrated, identified, and conceptualized as emotion or pain (Corder, 2019; Slaby, 2019). The role of the therapist in mediating the patient's arousal and the mood is essential, which is emphasized by research showing that arousal and N.A. are co-regulated by both the patient and the social environment (Barrett, 2014; Krueger, 2016; Slaby, 2019). Different attentional strategies such as Distraction (Fox and Calkins, 2003; Rothbart and Derryberry, 1981) and affect-biased attention (Morales et al., 2016) have been shown to decrease N.A. (Posner et al., 2012, 2014; Gross et al., 2011; Gyurak et al., 2011; Bronson, 2000; Todd et al., 2012; Todd and Anderson, 2013). Distraction is a cognitive emotion regulation strategy and refers to diverting attention away from N.A. by performing a working memory task (Koch et al., 2018; Gross et al., 2011; Gyurak et al., 2011; Fox and Calkins, 2003; Bronson, 2000). It is a self-regulation technique of shifting the attention away from the unpleasant internal or external stimuli to reduce negative affect. Harman et al. (1997) stated that for infants, a distraction for 10, 30, or 60 s is equally soothing. She also found a resurgence of distress expression after the Distraction.

ReAttach is another psychological intervention, in which environmental affect and arousal regulation, multiple sensory stimulations, and social cognitive training are combined (Weerkamp-Bartholomeus, 2018). Practical evidence reveals that ReAttach is a gentle and accessible learning intervention that reduces psychological distress and enhances learning conditions (Weerkamp-Bartholomeus, 2015, 2018). During ReAttach, the therapists provide tactile stimuli by gently tapping on the palms of the patient's hands. Some data demonstrated that low intensity, non-noxious activation of sensory nerves in the skin triggers oxytocin release (Uvnäs-Moberg et al., 2015; Walker et al., 2017). These findings suggest that perhaps even during ReAttach, by low intensity, non-noxious tactile activation of the hands, the natural bodily release of oxytocin, with its calming and relaxing effect, could be triggered.

W.A.R.A. is a sub-element of ReAttach, explicitly aiming at wiring unpleasant feelings to a sizeable neural ensemble, actively composed by the therapist through simultaneous activation of multiple concepts under ReAttach conditions (Weerkamp-Bartholomeus, 2019).

This pilot study aimed to investigate the efficacy of W.A.R.A. on N.A. in a single-blinded randomized controlled setting versus Distraction in different neuropsychiatric conditions.

## 2 Subjects and methods

### 2.1 Study design

We used a single-blind, randomized controlled, crossover equivalence design, to compare the efficacy on N.A. regulation of W.A.R.A. versus Distraction in Dutch patients with different neuropsychiatric disorders. The group of patients experienced problems in daily life functioning, such as dealing with stressful events, self-regulation, and executive functioning. The data were sampled as part of care as usual in Dutch clinical therapeutic settings, and therefore permission from the medical ethics review committee was not required. The trial was carried out by the ReAttach Therapy International Foundation in the Netherlands.

A priori power analysis was performed in G. Power (Faul, 2007) to calculate the study's required sample size. To detect clinically significant differences in the outcome (O.C.) measures with 80% power and an expected effect size of 0.30 (α = .05 two-sided) and presuming a drop-out rate of 1% a minimal sample size of 95 was required. We strived to recruit a sample of 90–100 patients in total.

Patients were 101 adult patients (76 women and 25 men, mean age 42.61 + SD 13.05 years) with neuropsychiatric disorders recruited by 13 qualified Dutch ReAttach therapists with a private practice (registered at reattachregister.org). Fifty-six patients had been diagnosed by a psychiatrist, (neuro)psychologist, or neurologist, as shown in Table 1. The remaining forty-six patients signed up for ReAttach treatment before diagnostics. In our sample, 21 patients used medication, as presented in Table 1. The medicines of all subjects were expected to remain stable during the study, due to the short study period (one therapy session).

**Table 1:** Sample description (N = 101) of diagnoses and medication.

Diagnoses	N
ADHD	4
Anxiety Disorder	4
Burnout	9
Cancer	1
Chronic Fatigue Syndrome	3
Chronic Pain	6
Depression	5
Diabetes	1
Eating Disorder	3
Functional Neurological Disorder	2
Inflammatory Bowel Disease	2
Obsessive Compulsive Disorder	1
Personality Disorder	3
PTSD	5
Sensory Processing Disorder	11
Tinnitus	1
Traumatic Brain Injury	3
Medication	N
Citalopram	4
Fluoxetine	2
Seroxat	5
Concerta	2
Ritalin	1
Strattera	1
Paracetamol	3
Diclofenac	2
Asacol	1

Dutch ReAttach therapists assessed eligibility, and we included adult patients who provided informed consent to participate in the study conducted at the time of the first ReAttach session. Exclusion criteria were reported suicidality risk and alcohol or drug abuse during the investigation.

#### 2.1.1 Randomization and intention to treat

The therapists were randomly assigned to group A (first Distraction and secondly W.A.R.A.) or B (first W.A.R.A. and secondly Distraction) with a random plan generator as followed: therapist 1, 5, 7, 8, 9 and 12 were assigned to group A, and 2, 3, 4, 6, 10, 11 and 13 to group B. Subjects could leave the study at any time for any reason without consequences.

#### 2.1.2 Blinding

**Figure 1:**
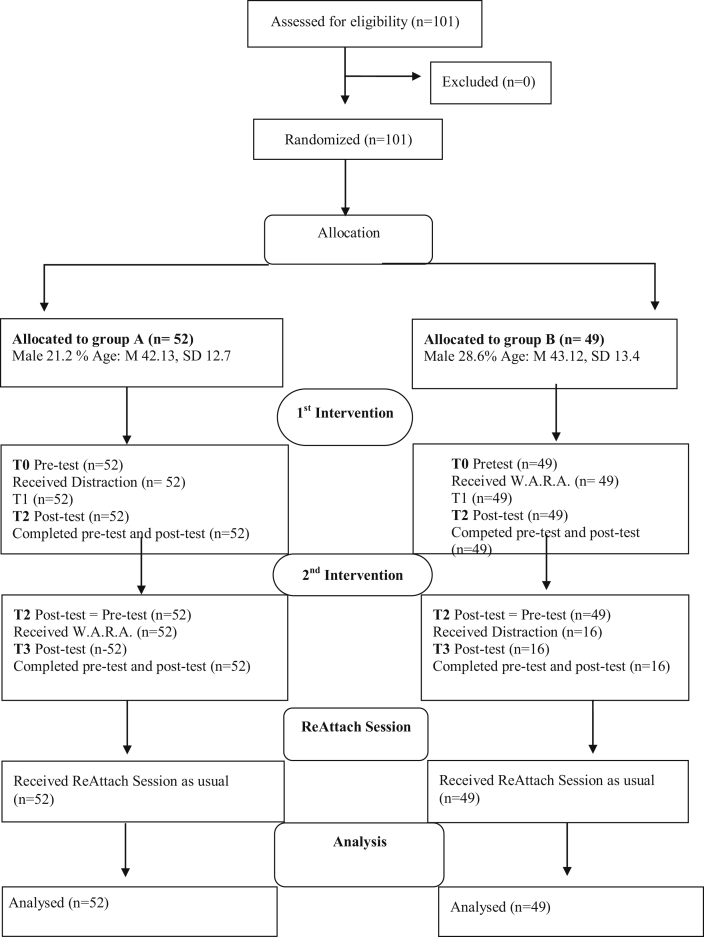
CONSORT diagram of study recruitment, treatment allocation and assessment including demographic characteristics.

Two self-administered questionnaires were designed including questions about demographics, therapist instructions, and questions about NA at different time points. One survey included instructions and measurements for group A (first Distraction and secondly W.A.R.A.) and another questionnaire for group B (first W.A.R.A. and secondly Distraction). All therapists received a personal hyperlink leading to the survey for either group A or B. The therapists and patients knew that Distraction and W.A.R.A. were offered as extra tools to regulate NA. They were blind to the sequence and comparison of both interventions. As a result of the randomization and anonymous data sampling, the researcher was blind to the assignment of patients. Figure 1 shows the flowchart of study recruitment, treatment allocation, assessment, gender and age. All the patients provided informed consent for anonymized data processing and participation in the research by an online agreement.

### 2.2 Interventions

Qualified ReAttach therapists (registered at reattachregister.org) provided W.AR.A. and received online instructions on how to provide Distraction. Psychopharmacological treatment was allowed since medications remained stable during this study, which lasted only one therapy session.

#### 2.2.1 Distraction

In this study, Distraction was offered as a self-regulation task to shift the attention away from an unpleasant feeling. All ReAttach therapists received the same online instructions for Distraction: The ReAttach therapist first instructed the patient to focus on the unpleasant feeling, and secondly asked the patient to distract him/herself by counting to ten. Since affect is co-regulated by the social environment (Slaby, 2019; Krueger, 2016; Barrett, 2014) the presence of the therapist played a role in the intervention outcome of Distraction. This co-regulation effect was counterbalanced for by randomization and the within-subjects design.

#### 2.2.2 W.A.R.A.

W.A.R.A. is designed for ReAttach therapists working with patients with chronic emotional dysregulation, sensory over responsivity, and chronic pain. Even though previous studies have shown that ReAttach reduces overlapping symptomatology in patients with emotional dysregulation and chronic pain (Ter Maat, 2018; Chauhan, 2018; Petter, 2018; Weerkamp-Bartholomeus, 2018), there were still patients with persistent complaints of hypersensitivity of intense, unpleasant feelings.

During ReAttach, we usually aim to influence these symptoms by social cognitive training under optimal sensory processing conditions. ReAttach offers patients the opportunity to train mentalization, to modify cognitive biases, and to use associative memory formation for active learning (Bartholomeus, 2013). The cause of and therefore, a possible solution for these persistent symptoms could lie in the fact that the regular ReAttach procedure went too fast for these patients, skipping a necessary step in the development of their healthy sensory stimulus processing. Reviewing the work of Slaby (2019) and Krueger (2016) on emotional and physical pain, the insight arose that the regular ReAttach procedure aimed to change concepts, namely the concept of pain and the concept of emotion, by identification with and adjusting them, and by fostering more adaptive coping styles. However, for patients with persistent complaints, it might be adequate to change our main focus to unpleasant feelings for which there are no words yet. In other words, with W.A.R.A., we address to pre-conceptual negative affect instead of working with concepts of pain or emotion. During the W.A.R.A. instruction, the therapist co-regulates negative affect, which is only referred to as an unpleasant feeling, by manipulating arousal and sensory stimuli. Immediately after the downregulation of the arousal, the therapist mentions five positive concepts that follow each other quickly and are not related but are grouped only for this W.A.R.A. exercise. During the downregulation, the patient needs to focus on the pre-conceptual unpleasant feeling and simultaneously associate on the presented conceptual ensemble. Subsequently, the patient receives an instruction for associative memory formation (remember this) during a few seconds of fast tapping. After a second downregulation, the patient integrates both the unpleasant feeling and the conceptual ensemble provided that the timing and co-regulation are optimal. In the period of the W.A.R.A. training courses, it became clear that therapists who failed to provide the group of positive concepts quickly enough after downregulation were unsuccessful. As previously stated by Donald Hebb (1949), timing is of significant importance: “Cells that fire together, wire together”. In Wiring Affect with ReAttach, this is undoubtedly true.

W.A.R.A. involved external affect regulation and arousal regulation by the therapist. At first, the therapist stimulated multiple senses: visual by facial expression and eye contact, auditive by verbal instruction or by making sounds and tactile by fast tapping on the back of the patient's hands. The therapist instructed the patient to close the eyes and focus on the unpleasant feeling during the complete exercise. Shortly after that, the therapist activated low arousal by the change of tapping frequency. During a low-frequency tapping, the therapist instructed the patient to focus on five positive concepts (such as enthusiasm, love, holiday, friends, excitement). Then the therapist changed the tapping speed again into fast tapping, to activate optimal arousal to process the next instruction: to remember the exercise so far. Immediately after this instruction, the therapist changed tapping speed again towards low arousal. During the low arousal, the therapist remained silent for 20 s. The therapist ended the W.A.R.A. exercise under fast tapping conditions by the instruction to open the eyes.

### 2.3 Procedure

At the beginning of the therapy session (T0), patients of both groups were asked to focus on NA and rate the intensity of unpleasantness on an 11 points numerical rating scale, commonly used to evaluate the severity of pain (Williamson and Hoggart 2005). Instead of referring to physical or emotional pain, we asked the patients to assess the unpleasant feeling on a scale of 0 (not unpleasant at all) to 10 (most unpleasant).

After the baseline rating, subjects from group A received Distraction, and patients from group B received W.A.R.A. Both interventions took approximately the same amount of time: between 60 and 90 s. Immediately after the first intervention (T1), the therapists asked the subjects if the unpleasant feeling was still present or gone. Descriptive statistics with percentage values of frequencies indicated that immediately after the intervention (T2), 20% of the subjects after Distraction, and 74% of the patients directly after W.A.R.A claimed that the unpleasant feeling was gone, as presented in Table 2. All patients were asked to refocus on the negative feeling to investigate if they could bring the feeling up again. Subsequently, all subjects were asked to rate the intensity of unpleasantness for the second time (T2).

**Table 2:** Comparison of immediate post intervention response (T1).

Immediate response	The feeling is gone	The feeling is not gone
Distraction (n = 54)	20%	80%
W.A.R.A. (n = 49)	74%	26%

After re-engagement with the feeling, 18.2% of the subjects who received Distraction as the first intervention and 26.1 % of the individuals who received W.A.R.A., stated that the unpleasantness was less intense or changed. As presented in Table 3 and 81.8 % of the subjects who received Distraction compared to 6.5 % of the persons who received W.A.R.A. reported no change at all. No patients in the Distraction group, as compared with 67.4% of the patients of the W.A.R.A. group, claimed after re-engagement that the unpleasant feeling was gone. If subjects could not bring the negative feeling up again, the NA was rated as 0. Patients who had lost the negative feeling could not follow through with the double task and partially dropped out.

**Table 3:** Comparison of post-intervention response after refocusing (T2).

After re-engagement	The feeling is gone	Less intense or changed	Hasn't changed at all
Distraction (n = 54)	-	18.2%	81.8%
W.A.R.A. (n = 49)	67.4%	26.1%	6.5%

**Figure 2:**
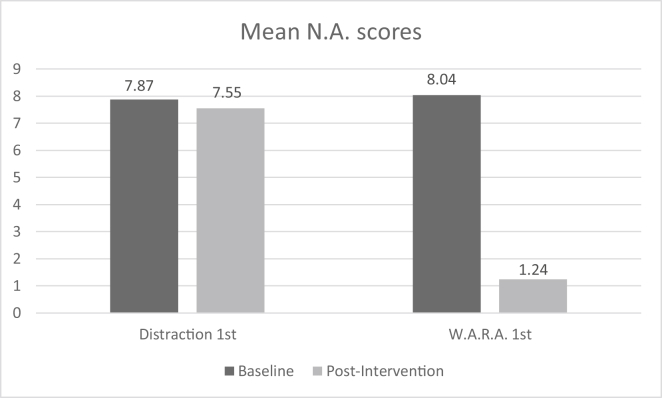
Mean baseline (T0) and post-intervention ratings (T2) on N.A. for Distraction as first intervention and W.A.R.A as first intervention.

Immediately after the second rating (T2), the remaining subjects from group A received W.A.R.A. as a second intervention, and the remaining subjects from group B received Distraction as a second intervention. After the second intervention, all subjects were asked to refocus on the negative feeling and to rate the intensity of unpleasantness for the third time (T3). The zero scores at T2 from subjects who partially dropped out were carried on to T3 due to the intention to treat procedure. Figure 2 represents the comparison of mean affect scores at baseline (T0) and post-intervention (T2) for Distraction as first intervention (M1 – 7.87, M2 = 7.55) and W.A.R.A. (M1 = 7.55, M2 = 1.24) as first intervention. After the third rating, all subjects continued the therapy with their first ReAttach session.

### 2.4 Data analysis

Descriptive statistics and a Consort Flowchart were used to contextualize the demographic characteristics of the study population. We used the Mann Whitney U Test, Wilcoxon Signed Rank Test, and one-way ANOVA for comparison of demographic characteristics and outcome measures between the groups at baseline. Mann-Whitney U test was used for comparison of intervention outcome between groups. A Friedman test was run to compare intervention for the within-subjects design. Pairwise comparisons were performed (SPSS Statistics, 2012). Effect-sizes for the intervention outcomes within-subjects were estimated by single group pre-test post-test design by taking the correlation between pre- and post-test into account (Morris, 2008, Morris and DeShon, 2002). All statistical tests were two-sided, and we set the significance level at 5%. We reported the interpretation of effect sizes conform Cohen (1988). The Statistical Package for Social Science (SPSS) version 22 (Armonk, NY, USA) (IBM Corp., 2012) was used to analyse the research data.

## 3 Results

### 3.1 Descriptive statistics

As shown in Figure 1, the distribution of age and gender were the same in groups A and B. One-way ANOVA was conducted to assess whether there were significant differences associated with pharmacological therapy. No differences were found between patients with medication (N = 21) and patients without medication (N = 80) in baseline N.A. rating. F(1, 99) = .022, p = .881 and after the double-task F(1,99) = .824, p = .366.

The Mann-Whitney U test indicated that there was no significant difference (Z = 0.574; p = .566) between group A (mean rank 49.50) and B (mean rank 52.79) at baseline NA rating.

### 3.2 Comparison distraction versus W.A.R.A.

**Figure 3:**
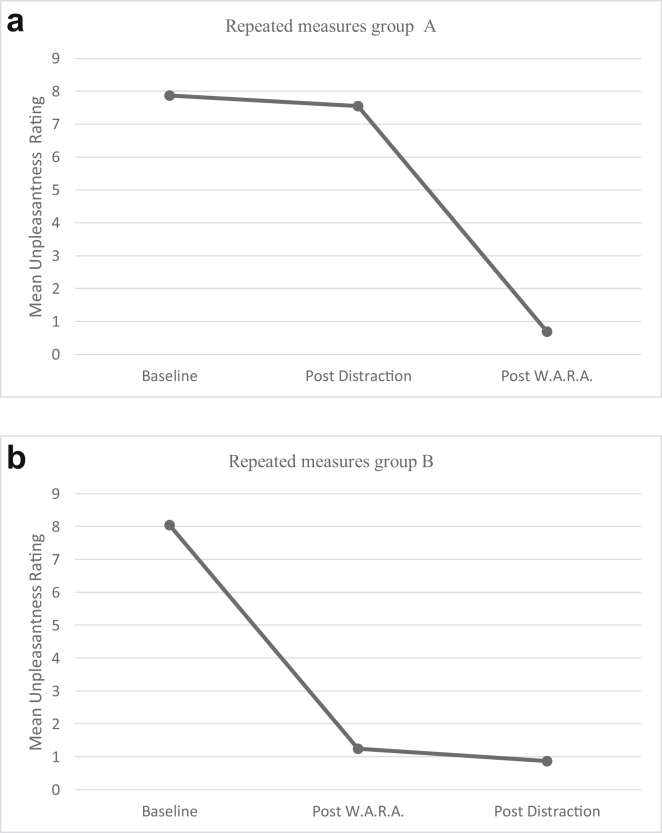
a: Repeated measures group A. b: Repeated measures group B.

Negative affect was significantly different at the different time points, X^2^(5) = 339.743, p < 0.005. Figure 2 presents the mean baseline (T0) and mean post-intervention (T2) scores NA for Distraction and W.A.R.A. as first intervention. We compared the intervention outcome (OC) at different timepoints by differences in NA-scores across group A and group B. The first intervention outcome (OC1) was significantly higher in group B (mean rank = 77.05, W.A.R.A.) than in group A (mean rank = 29.21, Distraction, U = 2463, z = 8.380, p < 0.05). The second intervention outcome (OC2) was significantly higher in group A (mean rank = 73.00, W.A.R.A.) than in group B (mean rank = 24.70, Distraction). There was no significant difference in the outcome of the double task (Distraction and W.A.R.A., U = 1258, z = -.045, p = .964) (Figure 3a,b).

The intervention outcome was statistically significantly different between the interventions, X^2^(2) = 193.729, p < 0.001. The post-hoc analysis revealed that W.A.R.A. (Mdn = 7.00) was statistically significantly more effective than Distraction (Mdn = .00) (p < 0.001), with a large effect size for W.A.R.A. (dRMpooled = 2.384, 95% Confidence Interval 2.521–3.069) and a medium effect size for Distraction (dRMpooled = 0.38, 95% Confidence Interval 0.183–0.577).

In group B only 13 subjects received Distraction. The NA had disappeared after W.A.R.A.; therefore, 33 out of 46 subjects could not bring the NA up again and did not receive Distraction as a second task. In the group A (N = 55) where Distraction was the first intervention, the mean difference between the pre-test (M = 7.97, SD = 1.54) and post-test (M = 7.15, SD = 2.50) was 0.72 and in group B (N = 46) where Distraction was offered as the second intervention the mean difference was 0.50 (M1 1.24, SD 2.18 and M2 0.74, SD 1.83).

The distribution of Distraction was similar in both groups, as assessed by visual inspection. The efficacy of Distraction was not significantly different between group A (Mdn = .00) and group B (Mdn = .00, U = 1206, z = -.507, p = .612), with medium effect sizes for group A (dRMpooled = 0.42) and group B (dRMpooled = 0.36) The distribution W.A.R.A. was similar in both groups, as assessed by visual inspection. The efficacy of W.A.R.A. was not significantly different between group A (Mdn = 7.00) and group B (Mdn = 7.00, U = 1275, z = .069, p = .945) with large effect sizes for group A (dRMpooled = 2.173) and group B (dRMpooled = 2.681). Evaluation of the intervention outcomes in terms of success (positive change) or failure (no change or negative change) resulted in a probability of success for W.A.R.A. of 95%. The likelihood of success for Distraction was 26.7%.

## 4 Discussion

The present study aimed to investigate the efficacy of W.A.R.A., an affect regulation strategy, based on disengagement (Gross et al., 2011; Gyurak et al., 2011; Fox and Calkins, 2003; Bronson, 2000), through a prospective single-blinded, randomized controlled, crossover trial, in a large sample of patients with different neuropsychiatric conditions. W.A.R.A. is an intervention of only ninety seconds, build up from the critical elements of ReAttach, a sensory and social cognitive integration training, on N.A. (Weerkamp-Bartholomeus, 2018, 2019). This study is the first to investigate the efficacy of W.A.R.A. as compared with Distraction, a psychological technique that is an effective emotion regulation strategy based on diverting attention away from N.A. by performing a working memory task (Koch et al., 2018).

The results of this study revealed that W.A.R.A. was significantly more beneficial with a large effect size (dRMpooled = 2.38), as compared with Distraction with a medium effect size (dRMpooled = 0.38).

After W.A.R.A., 89% of the subjects reported that the N.A. was less intense/had changed (26.1%) or was gone (67.4%). We found a carryover order effect after W.A.R.A. as the first intervention, but not after Distraction as the primary intervention. Therefore, a secondary outcome of the study was that, in contrast to W.A.R.A., the effect of Distraction was temporary. Re-engagement with the N.A. after the distractive task caused a revival of the unpleasant feeling in all patients. After re-engagement, no subject claimed that the negative feeling had disappeared after Distraction. A vast majority of the Distraction patients (82 %) reported that after re-engagement, the N.A. had not changed at all. The remaining Distraction patients (18.2%) indicated that the intensity or the feeling had changed in the meantime. Internal or external change in arousal might have caused these changes in intensity since arousal was co-regulated by the presence of the therapist.

It is not taken for granted that patients agree to the tapping on the hands. W.A.R.A. requires proximity to the therapist and the patient's trust. Before any treatment can be offered, the ReAttach therapist will have to invest in a good working relationship with the patient. It is common practice and necessary to provide basic information about ReAttach and individually pay attention to the multiple sensory stimulations, the arousal regulation, and to the associative nature of ReAttach. Since W.A.R.A. consists of the essential elements of ReAttach, it makes the exercise particularly suitable as a first introduction of the ReAttach intervention. Therefore, W.A.R.A. is scheduled before the start of the first ReAttach session.

In this study, all participants went along with the tactile stimulations, probably due to the explanation of the intervention, and the predictability of the touch. It is an interesting phenomenon that W.A.R.A. can be used to reduce tactile over-responsivity. Patients with resistance to the proximity of the therapist or to physical contact, receive self-regulation exercises. W.A.R.A. can be provided as a self-regulation tool. This study focuses exclusively on W.A.R.A. provided by the therapist. Another research is currently investigating the efficiency of W.A.R.A. provided as a self-regulation tool versus W.A.R.A. face to face by the therapist.

The conceptualization of negative affect in unpleasant feelings may still be a point of discussion. During the W.A.R.A. instruction, language is essential. The therapist is instructed not to visualize or conceptualize the unpleasant feelings for the patient to be able to address pre-conceptual negative affect: it is only referred to as an unpleasant feeling. The grouped words needed to build a temporary ensemble of concepts may be randomly chosen. To make W.A.R.A. a pleasant intervention, the therapists are instructed to select positive concepts that fit the patient's world of experience.

Besides W.A.R.A., that in the current study showed a superior outcome effect compared to Disengagement, Eye Movement Desensitization and Reprocessing (E.M.D.R.) therapy, which is similar to Disengagement in terms of taxing the working memory, also proved to be effective in terms of reducing N.A. (Van Etten and Taylor, 1998; Bradley et al., 2005; Davidson and Parker, 2005). The taxing of working memory for both W.A.R.A. and E.M.D.R. therapy might be different. W.A.R.A. provides external arousal regulation, multiple sensory stimulations, and mentalization to active generations by the subject. E.M.D.R. therapy does this by a standard protocol that includes eight phases and bilateral stimulation (usually horizontal saccadic eye movements) to reduce N.A. caused by traumatic memories (Shapiro, 2005). Therefore, E.M.D.R. is a strategy requiring long-term sessions, while W.A.R.A. is shorter, more cost-effective, and, as such, with better patients' compliance. The most essential difference between W.A.R.A. and E.M.D.R. is that W.A.R.A. (and ReAttach) is not trauma focused. In any case, future studies comparing the efficacy of W.A.R.A. versus E.M.D.R. therapy in terms of N.A. reduction and regulation would be desirable.

The present study suffers from several limitations that should be acknowledged. First, the heterogeneity of the research population makes it challenging to interpret the study outcomes and to give clear recommendations for specific patient groups. Second, we used a Numeric Rating Scale, initially designed for the evaluation of pain (Williamson and Hoggart, 2005), to rate the intensity of unpleasantness. Although this rating scale provided continuous data, these were not normally distributed: this might have prevented the detection of the increase of N.A. when the baseline scores were high. Furthermore, we have not assessed to what degree the reduction of N.A. was clinically relevant in the broader psychopathological context. This requires further investigation. The presence of a therapist played a role in the outcome of both W.A.R.A. and Distraction since affect is co-regulated by the social environment (Slaby, 2019; Krueger, 2016; Barrett, 2014). This co-regulation effect was counterbalanced by randomization and the within-subjects design. As in any psychotherapy, placebo effects likely played a role in both interventions (Enck and Zipfel, 2019). W.A.R.A. and Distraction are not comparable in the intensity of contact with the therapist, and the expectation-inducing effect of W.A.R.A. may be more powerful. The fact that W.A.R.A. involves touch and Distraction not may also influence the results. Further research should examine these contributing factors more in detail.

## 5 Conclusions

Although carried out in a single-blinded, controlled design, the study must be considered to be a pilot. Nevertheless, the results would suggest that W.A.R.A. may be an effective, accessible, and short transdiagnostic intervention for the reduction of N.A. in different neuropsychiatric disorders. Again, W.A.R.A. seemed without short-term revival after re-engagement. As compared with Distraction, a self-regulation exercise with a temporarily medium effect, W.A.R.A. was found to be significantly more beneficial. However, our findings require to be replicated in larger samples of patients with specific neuropsychiatric conditions, and/or to be corroborated by follow-up data to explore how long W.A.R.A. effects might last. Our opinion is that W.A.R.A., and other psychological techniques if substantiated by further data gathered in controlled trials, would constitute accessible, gentle, and evidence-based transdiagnostic interventions for specific symptom clusters present in a wide range of different neuropsychiatric conditions.

## Declarations

### Author contribution statement

P. Weerkamp-Bartholomeus: Conceived and designed the experiments; Performed the experiments; Analyzed and interpreted the data; Contributed reagents, materials, analysis tools or data; Wrote the paper.

E. Chan, A. Srivastava: Conceived and designed the experiments; Wrote the paper.

D. Marazziti, T. Van Amelsvoort: Analyzed and interpreted the data; Wrote the paper.

### Funding statement

This research did not receive any specific grant from funding agencies in the public, commercial, or not-for-profit sectors.

### Competing interest statement

The authors declare the following conflict of interests: P. Weerkamp-Bartholomeus designed both ReAttach and W.A.R.A.

### Additional information

No additional information is available for this paper.
